# Comparison of implant success versus endodontically treated teeth: A retrospective cohort study

**DOI:** 10.6026/973206300222229

**Published:** 2026-04-30

**Authors:** Monika Rawat, Ashish Maheshwari, Soumadip Dey, Himanshu Sharma, Sovendu Jha, Nadira Sultana

**Affiliations:** 1 Department of Conservative Dentistry and Endodontics, SMIH, Dehradun, Uttarakhand, India; 2 Department of Dentistry, Gajra Raja Medical College, Gwalior, Madhya Pradesh, India; 3 Department of Oral & Maxillofacial Surgery, Awadh Dental College & Hospital, Jamshedpur, Jharkhand, India; 4 Department of Oral & Maxillofacial Surgery, Maharishi Markandeshwar Dental College Sciences & Research, Maharishi Markandeshwar (Deemed To Be University), Mullana, Ambala, Haryana, India; 5 Department of Orthodontics and Dentofacial Orthopaedics, Buddha Institute of Dental Sciences and Hospital, Patna, Bihar, India

**Keywords:** Dental implants, endodontic treatment, root canal therapy, survival rate, success rate, tooth preservation

## Abstract

Choosing between endodontic preservation and implant replacement for compromised teeth poses key clinical challenges for outcomes and costs. Hence, this retrospective cohort study compared 198 implants and 214 endodontically treated teeth at a university hospital (2018- 2020) over five years. Success rates reached 89.3% for endodontically treated teeth and 93.4% for implants (p=0.142), with survival at 94.9% and 96.5% respectively (p=0.423). Complication rates were comparable overall, though types differed between the two modalities. Thus, both treatments offer excellent long-term predictability, supporting endodontic therapy as a viable alternative to implants for suitable teeth.

## Background:

Conservation of natural dentition has in the past been regarded as the main goal of dental care and extraction was the last resort when teeth were no longer restorable [1]. Nevertheless, the incredible changes in the field of implant dentistry in the last forty years have utterly changed this paradigm, giving clinicians and patients a more stable alternative to preserving the damaged teeth [2]. This has caused a lot of controversy on the proper signs and symptoms of each course of treatment and the long-term results that patients are likely to get. The root canal therapy, which is part of endodontic treatment, is still one of the most common dental treatments performed globally at a success rate that has been reported to be between 85 and 97% according to the standards and the types of cases that have been included in it [3]. The contemporary endodontic procedures, such as the use of operating microscopes, nickel-titanium rotary instrumentation and sophisticated obturation, have greatly enhanced predictability in the treatment [4]. Furthermore, there has been the eventual expansion of the scope of conditions that can be treated to bioactive endodontic materials and regenerative procedures. At the same time, dental implant therapy has ceased to be an experimental treatment whose success is documented in a few cases, but rather has become main stream. Current literature documents implant survival rates of over 95 per cent at a decade and also that the success rates of implantation are nearly the same as those of natural tooth retention [5]. The further development of implant outcomes and decreased complexity of treatment has been achieved by the introduction of computer-guided surgery, surface modification and the development of prosthetics [6]. A choice between these two treatment options does not exclude various factors that need to be taken into account, such as the clinical manifestation of the disease, patient preferences and the cost, as well as the prognosis in the long term [7]. Whereas the implants have the benefit of complete removal of the diseased tooth structure, the endodontic treatment has the benefit of preserving the proprioceptive function of the periodontal ligament and retaining the natural architecture of the alveolar bone [8]. Moreover, financial aspects also vary considerably, as the implant therapy is a much higher initial cost. Recent systematic reviews and meta-analyses have tried to come up with a synthesis of the available evidence between these modalities of treatment [9]. Nevertheless, the heterogeneity in methods, a difference in the criteria of success and dissimilarity in follow-up time has complicated direct comparisons. Studies have proposed similar numbers of implants and endodontically treated teeth, whereas others have shown one method as being superior compared to the other in certain clinical conditions [10]. One key point of criticism in this comparison is the meaning of success and survival. Whereas survival only implies that the tooth or implant is in operation, success has other elements such as no symptoms, radiographic pathology and complications [11]. This difference is specifically applicable when considering the quality of results and not just the length of treatment. There are also fundamental differences between the biological complications of each of the treatment modalities. Endodontically treated teeth can have a history of periapical pathology, vertical root fracture or coronal leakage, whereas implants are prone to peri-implantitis, mechanical failures and aesthetic problems [13]. It is important to understand the nature and occurrence of such complications to be able to plan treatments and advise the patient on the same. Although the research in this field has been done extensively, there is a large gap in the research with respect to a direct comparison of these treatments in similar clinical conditions in the same group of patients. Numerous other studies that exist compare heterogeneous groups with varying baseline characteristics, which restricts the validity of the findings made [13]. Moreover, the impact of confounding factors, including the tooth location, the presence of an underlying pathology and systemic factors in the patient, should be put into consideration. Therefore, it is of interest to relatively compare the success rates, survival rates and complication profile of dental implants and endodontically treated teeth with a matched population of patients assessed over a five-year follow-up using standardised assessment criteria and controlling all pertinent confounding variables.

## Materials and Methods:

## Study design and setting:

This is a retrospective cohort study that was done at the University Dental Hospital under approval of the Institutional Ethics Committee (Protocol Number: IEC/DENT/2023-142). The research was carried out in relation to the STROBE principles of observational research. Endodontics and Oral Implantology departments of the hospital searched through the patient records from January 2018 to December 2020.

## Sample size calculation:

The power analysis software was used to estimate the sample size by assuming that the difference in the success rates between the groups is 5%, where the values of alpha are 0.05 and power=0.80. A sample size of 180 subjects per group was taken to be the minimum required. Considering the possible incompleteness and loss of data to follow-up, a group of 200 patients was set as a target.

## Population and selection criteria of the study:

The inclusion criteria were as follows: the age of the patients included in the study was between 18 and 70 years, the intervention was a single-tooth treatment (either endodontic therapy or single implant placement), the minimum period of follow-up was 60 months and the patients received treatment administered by highly qualified practitioners (specialists or residents under direct supervision).

## Inclusion criteria included:

Patients who had not received bisphosphonate therapy or immunosuppressant's within 12 months; patients who smoked more than 10 cigarettes a day; teeth that needed retreatment following a previous endodontic treatment; immediately loaded implants; incompleteness of documentation; and non-attendance of patients at follow-up visits.

## Group allocation:

Patients were divided into two groups according to the treatment they received:

[1] Group A (Implant Group): Patients who had briefly extracted teeth and were given single dental implants. The implants were all positioned in accordance with normal delayed procedures (minimum 3 months after extraction) and cemented or screw-retained crowns were positioned after a 3-6 months period of the implant being positioned.

[2] Group B (Endodontic Group): Patients who were treated with the primary root canal therapy for pulpal or periapical pathology. All the treatments were done by modern techniques, rotary nickel-titanium instrumentation and warm vertical condensation obturation. Final restoration using either direct composite restorations or indirect full coverage crowns was done within 4 weeks of endodontic therapy.

## Data collection:

Patient records were used to collect demographic and clinical data, including: age, sex, medical history, tooth/implant position, presence of preoperative periapical pathology, restoration type and all clinical observations at follow-up visits. Two calibrated examiners were used to assess radiographic data of the preoperative, immediate postoperative and follow-up periapical radiographs.

## Outcome measures:

[1] Primary Outcome - Success Rate: In endodontically treated teeth, the modified criteria of success were: no pain or swelling; no sinus tract; normal function; no finding of tissue loss on radiograph; radiographic signs of healing or stable periapical condition. In the case of implants, success was determined as follows: no clinically significant mobility; no persistent pain or dysesthesia; no radiolucency around implants; no suppuration or bleeding on probing more than 3mm in depth; and a satisfactory aesthetic outcome.

[2] Secondary Outcomes: These were the survival rate (tooth/implant that is working in spite of complications), complication rates and the types of complications experienced.

## Radiographic evaluation:

The scoring system of the Periapical Index (PAI) of endodontically treated teeth was used to assess periapical radiographs. The marginal bone level changes were evaluated with the help of the implant radiographs, which were estimated between the implant platform and the first bone-implant contact point. Calibrated digital software that had magnification abilities was used to carry out measurements.

## Statistical analysis:

The statistical analysis was conducted in SPSS version 26.0 (IBM Corporation, Armonk, NY, USA). Standard deviations, percentages, frequencies and means were all included as descriptive statistics. Categorical variables were compared between groups with Chi-square tests or Fisher-Precise tests depending on the situation. Independent t-tests were used to compare the continuous variables after the existence of normal distribution was checked by the Kolmogorov-Smirnov tests. Kaplan-Meier survival analysis was done to estimate cumulative survival and log-rank between-group comparison. The multivariate Cox regression analysis was conducted to establish independent predictors of failure. The p-value of statistical significance was considered to be p<0.05.

## Results:

A total of 412 patients met the inclusion criteria and were included in the final analysis. The implant group comprised 198 patients (48.1%), while the endodontic group included 214 patients (51.9%). Baseline demographic and clinical characteristics are presented in Table 1 (see PDF). The groups were comparable in terms of sex distribution, tooth position, arch distribution and follow-up duration. The implant group was significantly older than the endodontic group (p=0.012). Success and survival rates for both treatment modalities are summarised in Table 2 (see PDF). The overall success rate was 93.4% (185/198) for the implant group and 89.3% (191/214) for the endodontic group. This difference was not statistically significant (p=0.142). Survival rates were 96.5% (191/198) for implants and 94.9% (203/214) for endodontically treated teeth (p=0.423). Subgroup analysis by tooth position revealed the highest success rates in anterior teeth for both groups, with decreasing success in premolars and molars. However, these differences were not statistically significant within or between groups. Complication rates and types are detailed in Table 3 (see PDF). The overall complication rate was 15.7% (31/198) for implants and 18.7% (40/214) for endodontically treated teeth (p=0.416). The nature of complications differed substantially between groups. In the implant group, the most common biological complication was peri-implantitis (6.1%), while in the endodontic group, persistent periapical pathology was the primary biological failure mode (8.4%). Vertical root fracture accounted for 54.5% (6/11) of tooth losses in the endodontic group. Kaplan-Meier survival analysis demonstrated no statistically significant difference in cumulative survival between groups (log-rank p=0.386). The 5-year cumulative survival probability was 96.2% (95% CI: 93.5-98.9%) for implants and 94.6% (95% CI: 91.6-97.6%) for endodontically treated teeth. Cox regression analysis identified the following independent predictors of failure: molar position (HR=2.14, 95% CI: 1.12-4.08, p=0.021), presence of preoperative periapical pathology in endodontic cases (HR=1.87, 95% CI: 1.05-3.34, p=0.034) and age over 60 years (HR=1.65, 95% CI: 0.89-3.06, p=0.112). Treatment modality was not an independent predictor of failure (HR=0.82, 95% CI: 0.48-1.41, p=0.476).

## Discussion:

The results of this retrospective cohort study prove that dental implants and endodontically treated teeth have similar success and survival rates in five years of observation. The 93.4 and 89.3 per cent implant and endodontically treated tooth success rates and the 96.5 and 94.9 per cent survival rates, respectively, are consistent with the modern literature and confirm the fact that tooth preservation is still a predictable form of treatment as long as it has been properly indicated [14]. These similar results in the present study contradict the growing tendency to believe that the use of implant therapy is simply associated with better long-term prognosis. The recent systematic reviews have also made similar conclusions that there is no notable difference in these treatment modalities with the use of strict inclusion criteria and standardised assessment protocols [15]. This observation is of great importance in terms of treatment planning since it indicates that aspects beyond the type of treatment could be more decisive than treatment type. The greater percentage of molar teeth in the implant group and the correlation of the molar position with the high risk of failure should be considered. The molars form a more complicated treatment situation for both treatment modalities, as they have a complicated root canal structure in endodontic and a greater occlusal load in implant cases [16]. The finding serves to highlight that case-specific factors are more important in predicting the outcome of treatments as opposed to generalisations based on the modality of the treatment. The trend in complications was completely different in the two groups since the biological and mechanical nature of each treatment method is entirely different. The most common complication was peri-implantitis, which was observed among the implant group and the cases were 6.1. This observation is in agreement with the recent articles that show that peri-implantitis occurs in 5% to 15% of the implants, based on the diagnostic criteria and the nature of the patient population [17]. The peri-implantitis chronic inflammatory process and the possibility of progressive bone loss are the serious issues of long-term implant maintenance. On the other hand, vertical root fracture was the most dreadful complication in the endodontic group since it bore over half of the tooth loss cases. This observation points out the structural weakness of endodontically treated teeth, especially those at the posterior positions with high occlusal force [18]. The preventive effect of full-coverage restorations in the prevention of such fractures has been long established and the observation that 38.3% of teeth endodontically treated only received direct restorations could have also contributed to this complication rate. The existence of preoperative periapical pathology proved to be a strong predictor for failure in endodontically treated teeth, which is in line with other research works that have reported a lower success rate in cases where the periapical disease is already established [19]. The observation highlights the significance of prompt intervention and indicates that results can be made the best by treating teeth where possible before they develop periapical lesions. Economically, the similar success rates reported show that the endodontic treatment is cost-effective as a first-line treatment in most clinical settings. Given that the cost of implant therapy is three to four times higher than that of root canal therapy with coronal restoration, the cost of treatment choice has significant financial consequences [20]. The analysis, however, failed to capture the cost of complications management or repair, which is also a significant consideration in a complete cost-effectiveness assessment. The age gap found between the two groups, with the knee-capped implant patients being much older in age, indicates the actual treatment trends in the real world, as tooth removal and the introduction of implants can be thought of as a more easily accessible option when it comes to the natural dentition becoming old-fashioned [21]. This discovery also reflects the possible confounding in the observational studies, which compare these forms of treatment, since the process of selecting patients may not be random. Some drawbacks of this research should be admitted. The retrospective type of design limits the possibility of managing all confounding factors and presents a risk of selection bias. The study might be limited to generalizability to other settings and populations due to the single nature of the research. Also, the five-year period of follow-up, although considerable in itself, might fail to identify the differences in long-term outcomes that can be seen after longer times of observation [22]. Another limitation is the heterogeneity of types of restoration in groups. Every implant was placed with full-coverage crowns and about a third of all endodontically treated teeth were direct-restored. Such a difference could have contributed to the complication profiles experienced in the endodontic group, especially with respect to root fracture occurrence [23]. The prospective studies in the future need to utilise longer follow-up and standard protocols so that their research can bring more conclusive results on comparative efficacies of these treatment modalities. Besides, patient-reported outcome measures such as quality of life and satisfaction should be included to offer a more robust evaluation of the success of treatment [24]. These findings have clinical implications to support the use of a patient-centred approach to treatment planning without the assumption of the superiority of either of the treatment modalities; instead, it considers the clinical, patient and economic-based factors in treatment planning of an individual. Endodontic therapy should be viewed as a main goal of treatment in cases where preservation of the natural teeth remains possible and only implant therapy should be used in those situations when preservation of the tooth is not possible or feasible [25].

## Conclusion:

Data shows dental implants and endodontically treated teeth achieve comparable 5-year success (93.4% vs 89.3%) and survival rates (96.5% vs 94.9%), with similar complication levels despite differing patterns. Molar position and preoperative periapical pathology predict failure, while treatment modality does not significantly affect outcomes. Endodontic tooth preservation proves a predictable, cost- effective alternative to implants when proper case selection considers clinical presentation, patient factors and preferences rather than assuming one modality's inherent superiority.

## References

[1] Borda MF (2025). Acta Odontol Scand..

[2] Sinsareekul C (2025). J Prosthet Dent..

[3] Georgios SC, Wolff LF (2026). J Dent..

[4] Afrashtehfar KI (2023). Evid Based Dent..

[5] Lee C-T (2022). J Periodontol..

[6] Varghese KG (2023). J Prosthet Dent..

[7] Selvaraj H (2023). BMC Oral Health..

[8] Wang W (2024). J Dent..

[9] La Monaca G (2021). J Prosthodont Res..

[10] Bezerra AP (2021). Clin Nutr..

[11] Li S (2024). Int J Oral Implantol (Berl)..

[12] Messias A (2023). J Clin Periodontol..

[13] Messias A (2023). Clin Oral Implants Res..

[14] Pachiou A (2025). J Prosthodont..

[15] Michaud PL (2025). J Dent..

[16] Atieh MA (2021). Cochrane Database Syst Rev..

[17] Afrashtehfar KI (2024). Evid Based Dent..

[18] Esposito M (2020). Clin Trials Dent..

[19] Berlin-Broner Y, Levin L (2020). Int J Oral Maxillofac Implants..

[20] Yuen JJX (2025). J Prosthet Dent..

[21] Majid OW (2024). Evid Based Dent..

[22] Patel SR (2024). Int Endod J..

[23] Meira IA (2022). J Prosthet Dent..

[24] Roper BM (2023). Int J Implant Dent..

[25] Di Francesco F (2021). BMC Oral Health..

